# Use of Fidji Cervical Cage in the Treatment of Cervical Spinal Cord Injury without Radiographic Abnormality

**DOI:** 10.1155/2013/810172

**Published:** 2013-06-17

**Authors:** Sheng-Li Huang, Hong-Wei Yan, Kun-Zheng Wang

**Affiliations:** Department of Orthopaedics, The Second Affiliated Hospital, School of Medicine, Xi'an Jiaotong University, Xi'an 710004, China

## Abstract

Spinal cord injury without radiographic abnormality (SCIWORA) is a rare condition seen in adults. Many interbody fusion cages have been developed for its treatment, but clinical studies of Fidji cervical cage are still scarce. A total number of five patients (four male and one female) were reviewed. The ages of the patients ranged from 40 to 60 years. All the patients underwent neurological and radiological examinations. Neurological and functional outcomes were assessed on the basis of Frankel's grade. Three of the patients were Frankel B, and the rest two were Frankel C. Magnetic resonance imaging was also performed for the evaluation of spinal cord and intervertebral disc injury. Anterior cervical discectomy and Fidji cervical cage fusion were performed for all. The fusion status was evaluated on the basis of X-rays. After surgical intervention, the clinical symptoms improved for all the patients. The disc interspaces in all the patients achieved solid union at final follow-up. Fidji cervical cage is very efficient in achieving cervical fusion in patients with SCIWORA. There are few complications associated with the use of this cage, and the functional and neurological outcomes are satisfactory.

## 1. Introduction

Spinal cord injury without radiographic abnormality (SCIWORA) is an uncommon traumatic myelopathy without evidence of vertebral fracture or malalignment on plain radiographs or computed tomography (CT). The cervical cord is most frequently affected. Although no radiographic abnormality is found in cervical SCIWORA, it is often associated with various grades of paralysis.

SCIWORA in adults has been reported in the literature as case reports or series [1, 2]. The treatment regarding this type of injury with either surgery or conservative measures remains controversial [3–6]. The surgical procedure of choice is commonly anterior cervical discectomy with interbody fusion (ACDF). This technique allows direct decompression of neural structures and stabilization of the affected motion segments.

The use of polyether ether ketone (PEEK) cages that are very popular in degenerative spine surgery has not yet been investigated in SCIWORA. Certainly, there are by now no studies about the practicability of Fidji cervical cage made of PEEK material in cervical SCIWORA. This paper presents our experience of using anterior cervical discectomy with Fidji cervical cage fusion in treating patients with cervical SCIWORA.

## 2. Materials and Methods

This study was approved by the Institutional Review Board at our institution. All patients provided informed written consent.

### 2.1. Patients

**Figure 1:**
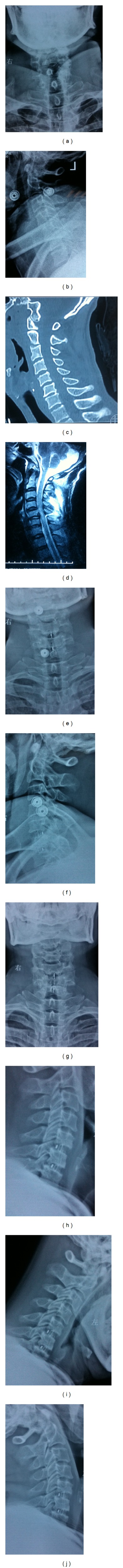
Example of a patient with SCIWOCTET. ((a), (b), and (c)) Preoperative plain radiographs and sagittal reconstruction CT scan of the cervical spine showing normal bone and alignment. (d) MRI demonstrating cord compression by herniated disc at the C5-6 level with cord edema. ((e), (f)) Plain films of the cervical spine on postoperative day 1. ((g)–(j)) Plain films of the cervical spine including lateral flexion (i) and extension (j) views at 3 months after surgery. There are bridging trabeculations across the disc space.

**Figure 2:**
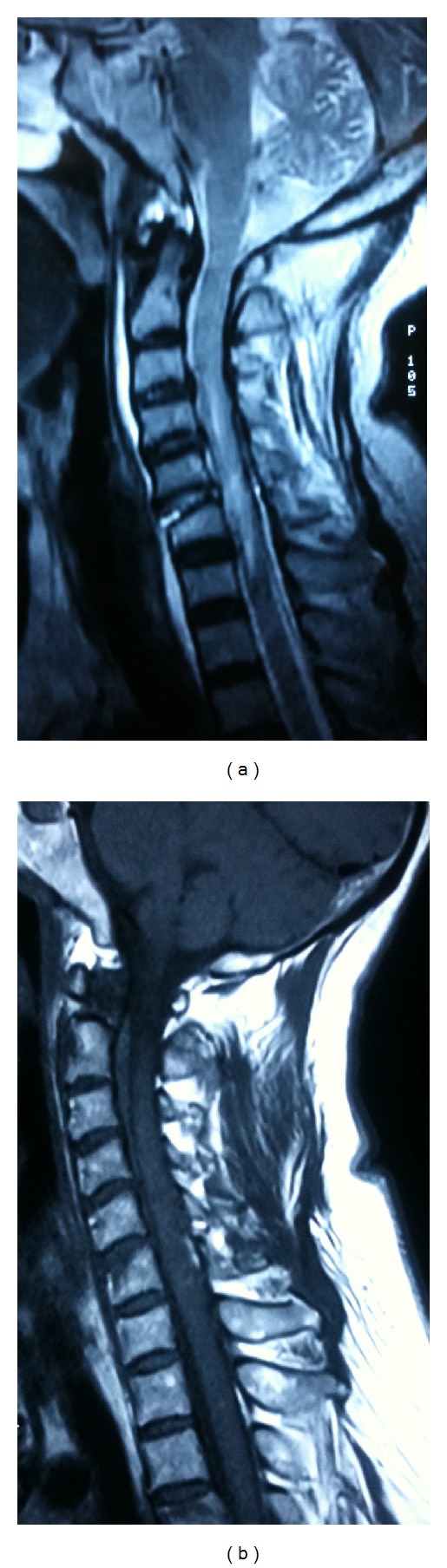
MRI of the patient. (a) T2-weighted image showing high signal intensity area at C5/6 intervertebral space. (b) T1-weighted image showing low signal intensity area at C5/6 intervertebral space.

All the patients with spinal cord injury treated at our department between January, 2008, and December, 2011, were reviewed. Those with fracture and/or dislocation, normal neurologic status, tumors, infections, deformity, rheumatoid arthritis, diabetes mellitus, previous cervical spine surgery, and neurodegenerative diseases were excluded. Finally, five patients were included, four males and one female, aged 40 to 60 years with a mean age of 48.8 years, who received treatment in our institution. The Frankel grade was used for the evaluation of the patients' neurologic status. Immobilization of the cervical spine was performed using a cervical collar before neurological and radiological examinations. The plain films and CT scans were normal—no fracture and dislocation. All the patients underwent magnetic resonance imaging (MRI) of the spine, which included T1- and T2-weighted sagittal and axial images. Initial cervical radiographic evaluation consisted of anteroposterior and lateral plain radiographs along with CT for suspicious regions. Dynamic views were not performed because of the immobilization. The diagnosis was established based on cervical X-ray films and CT images. In patients with neurological symptoms, MRI demonstrated intradiscal high signal intensity and/or disc herniations, accompanied with cord edema (Figures 1 and 2).

The patients had sustained injury at different parts of the cervical spinal cord but mostly at the C5-6 level, which was seen in four patients (80%). All the patients had evidence of cervical spine instability such as disc injuries. MRI revealed disc lesions. Three patients underwent a one-level fusion (two at C5-6 and one at C3-4), and two patients received a two-level fusion (one at C5-6 and C6-7, one at C4-5 and C5-6).  Table 1 summarizes the gender, age at the time of injury, level of vertebral injury, the period from injury to operation, disc herniation, and cause of injury.

### 2.2. Surgical Procedure

The surgery was performed under general anaesthesia using a standard right-sided anterior cervical approach. The patient was placed on a surgical bed in the supine position with the neck extended slightly. After fluoroscopic confirmation of the affected cervical level, a complete discectomy and decompression were performed. The discectomy was carried out using curettes and rongeurs, without a microscope or a high speed drill. The disc was completely removed, and the subchondral cartilage was carefully resected for the exposure of the cortical bone. Spondylotic spurs and herniated disc fragments were removed to decompress the spinal cord. An optimal Fidji cervical cage was selected following the completion of discectomy and endplate preparation. The inner cavity of the cage was filled with autologous cancellous bone. A wound drain was always placed in the neck. All the patients had worn cervical collars for three months. All the operations were done by the same surgeon.

The cancellous iliac crest autograft was harvested with a T-shaped driver. The skin wound of the harvest site over the iliac crest was 1 cm in length. The cancellous bone was cored out from the iliac crest between the inner and outer tables to 1.5 to 2 cm in depth by twisting the inner tube of the T-shaped driver.

### 2.3. Fidji Cervical Cage

**Figure 3:**
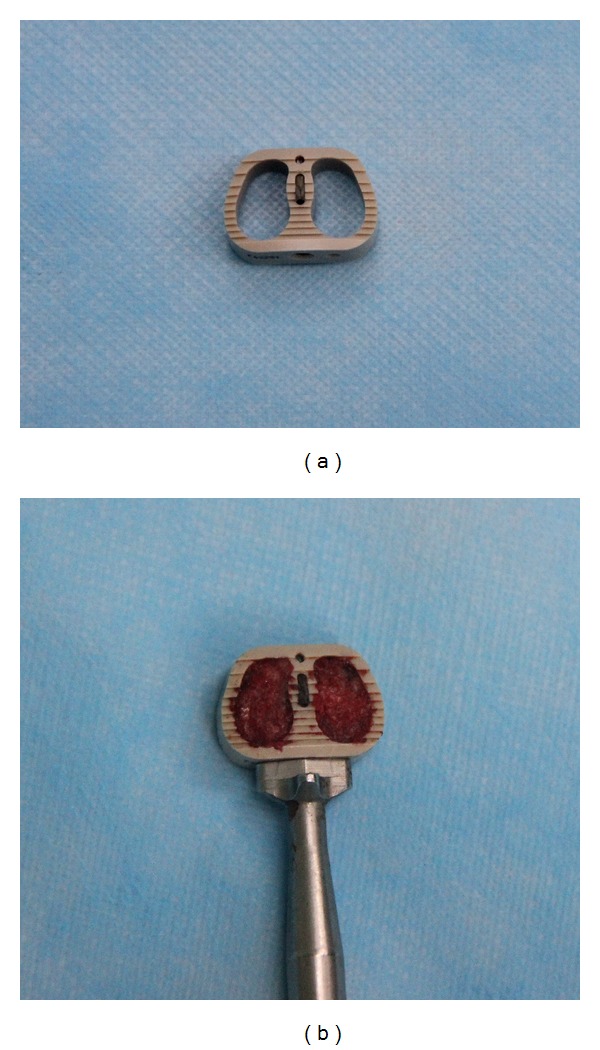
Photograph of a Fidji cervical cage. (a) Fidji cervical cage with halo frame. (b) Fidji cervical cage filled with autogenous cancellous iliac bone.

Fidji cervical cage (Abbott Spine, Bordeaux, France), made of PEEK material, is available in various height, and widths. It is horseshoe-shaped in axial section, containing two hollow cylinders filled with bone graft (Figure 3). In sagittal section, it is trapezoid shaped to provide a degree of lordosis. There is a retentive tooth on the top and the bottom, respectively, which improves the fixation of the cage to the bone. The two teeth are placed vertically in the medial plane and inserted 1 mm into adjacent vertebral bodies. As a result, Fidji cervical cage has a hard frame that resists spinal loading and maintains spinal alignment.

### 2.4. Outcome Assessment

Postoperative radiography was performed to all the patients to evaluate fusion and assess graft-related complications. Anteroposterior and lateral radiographs were taken immediately after the surgery and were then taken at intervals of 3, 6, 12, and 24 months. Union of the cage was defined as no radiolucent line between the cage and endplate and no translation at plain radiographs. Fusion was defined by continuous bridging of trabecular bone and the absence of radiolucent lines at 3 months after surgery on plain radiographs.

The patients were followed up at 3-, 6-, 12-, and 24-month intervals. By the time this research was conducted, all the patients had a minimum of one-year follow-up. Clinical outcome was assessed according to the Frankel grade. The criterion for significant improvement was a one-level rise in the Frankel grade. Successful treatment was defined as the patient achieving improvement in neurological status.

## 3. Results

### 3.1. Surgery

In this series, the mean operation time was 87 min (range, 70 to 118 min), and the estimated blood loss was below 40 mL in all patients.

### 3.2. Postoperative Course

All the patients had an uneventful postoperative course. No postoperative complications, including cerebrospinal fluid leakage and infection, were encountered, and no temporary neurological deterioration was observed. Neurological improvements were noticed immediately following surgery.

### 3.3. Follow-Up

All the patients had a regular follow-up ranging from 1 to 5 years (mean of 2.8 years). No cage subsidence or migration occurred, and no complications associated with bone graft were found. Complete interbody fusion was achieved in all cases. Intervertebral disc height was maintained throughout follow-up (Figure 1).

The preoperative and postoperative neurological status was compared (Table 2). Overall assessment at the time of admission and at 6 months after surgery revealed that all the patients made good recovery in neurological function. Patients with partial injury, which was represented by good Frankel grade, improved significantly within 6 months after surgery, and their Frankel grades remained stable during the follow-up.

## 4. Discussion

Cervical SCIWORA is an uncommon but destructive injury. The incidence of SCIWORA is about 10% of all spinal injuries [1, 7]. Road traffic accidents and falls are the two most common causes of SCIWORA, and it is especially falls in China [8]. The commonly practiced surgical treatment for this lesion is ACDF. To the best of our knowledge, the present study is the first to report the use of Fidji cervical cage in SCIWORA. Our results demonstrate the safety of the cage and significant improvement in patients. Those with partial injury with high Frankel grades may improve significantly shortly after surgery. 

There is some confusion about the nomenclature and definition of this type of injury. Pang and Wilberger [9] were the first to coin the acronym SCIWORA, which was before the advent of CT and MRI. By definition, SCIWORA consists of negative radiologic findings with plain radiographs and CT scans but not MRI. Different terminologies have been used to describe such an injury, for instance, cervical spinal cord injury without bone and disc injury [3], cervical spinal cord injury without bony injury [10], spinal cord injury without radiologic evidence of trauma [11, 12], occult cervical spine injury [13], and so forth. These terms are technically not accurate. In adult patients with this type of injuries, SCIWORA is actually a misnomer since the degenerative changes are actually radiographic abnormalities and/or accompanied with disc injury. Therefore, we suggest that this lesion should be called “spinal cord injury without bone injury and dislocation,” which we think most accurately describes the pathology of this type of injury.

MRI is of paramount importance for the evaluation of spinal cord damage, which has received considerable attention in the setting of acute trauma. It provides adequate information about neural and extraneural injuries and identifies treatable conditions. With the advent of MRI, the visualization of subtle lesions may reveal occult injuries in adults, such as SCIWORA. Disc abnormality after trauma is reported to range from 16% to 48% in the SCIWORA patients [7, 13–15]. An intervertebral space abnormality is suggestive of possible injury of the intervertebral disc that can be clearly visualized by MRI. For a disruption, the disc injury is best seen on sagittal T2WI as high signal intensity in the intervertebral site and low signal intensity on sagittal T1WI. As a result, MRI plays an important role in the acute posttrauma diagnostic and management decision-making processes. All the five patients reviewed in this study had disc interspace disruption, and each of them had positive MRI findings which correlated with their symptoms. Prolapsed intervertebral disc was noted in 4 (80%) cases.

It was reported that a considerable proportion of the patients with traumatic cervical SCIWORA had cervical segmental instability at the early stages of the injury [16]. The disc could be readily separated from the adjacent vertebral body on blunt dissection. This indicates a separation between the disc and the associated vertebra [17]. The goal of surgery is the reconstruction of the spinal column to achieve an improvement in the paralysis. ACDF is an effective surgical procedure for treating patients with SCIWORA. The quality of fusion depends mainly on the properties of the interbody graft and the graft placement technique. The choice of the graft material is therefore of critical importance. Fidji cervical cage is made of PEEK material, which is a semicrystalline linear biopolymer. The PEEK cages are biocompatible, non-absorbable, radiolucent, and corrosion resistant, and they have modulus of elasticity similar to the bone [18, 19]. In addition, the absence of cytotoxicity and mutagenicity in the PEEK cages has been demonstrated in an in vitro study [20]. In a word, Fidji cervical cages have the advantages of osteoconductive properties, good resistance to collapse, simplicity of use, and a physiological shape.

There is no relevant destruction of the adjacent vertebral bodies and endplates in SCIWORA, so a Fidji cervical cage used alone is firm and sufficient. The disc can be replaced by a Fidji cage without additional internal fixation. The surgical procedure is less complex and saves time. However, postoperative external immobilization is certainly necessary in the absence of a plate, and a hard cervical collar is advised for daytime wear. Fidji cervical cage is packed with iliac crest autograft in order to form a complete bone bridge between the endplates. In all of our patients, solid fusion was achieved. The cage has an identical surface area and does not need a drill for the preparation of the endplates, which avoids the cage from subsiding. As a result, the cage can provide stability, high fusion rates, and low subsidence. In addition, with radiolucency, Fidji cervical cage does not produce artefacts on radiographs or CT scan, which makes it easy to evaluate fusion status. In the cases reviewed by us, all the patients benefited from discectomy and Fidji cervical cage. No graft-related complications occurred. Fusion was also observed in the patients who had undergone a two-level discectomy.

## 5. Conclusion

Fidji cervical cage can be a safe and effective graft in treating cervical SCIWORA. However, the number of cases included in this study is limited, because it is very difficult to obtain an appropriate number of subjects, especially for a randomized clinical trial. Prospective clinical studies with greater numbers of cases should be conducted to verify the efficacy of Fidji cervical cage.

## Figures and Tables

**Table 1:** Demographic data of patients.

Patient	Gender	Age (year)	Affected level	Disc herniation	Time of injury (day)	Cause of injury
1	M	42	C5–7	+	4	Fall from height
2	M	40	C3-4		7	Fall down steps
3	M	45	C5-6	+	6	Motorcycle accident
4	F	57	C4–6	+	9	Fall down steps
5	M	60	C5-6	+	7	Fall

M: male; F: female.

**Table 2:** Frankel's grades at admission and at 6 months after surgery.

Patient	At admission	At 6 months operation	Follow-up (year)
1	B	D	2
2	B	D	4
3	C	E	1
4	B	D	2
5	C	E	5
